# Potent Cytotoxicity of Four Cameroonian Plant Extracts on Different Cancer Cell Lines

**DOI:** 10.3390/ph13110357

**Published:** 2020-10-31

**Authors:** Ahmed Somaida, Imran Tariq, Ghazala Ambreen, Ahmed Mohamed Abdelsalam, Abdallah Mohamed Ayoub, Matthias Wojcik, Jean Paul Dzoyem, Udo Bakowsky

**Affiliations:** 1 Department of Pharmaceutics and Biopharmaceutics, University of Marburg, 35037 Marburg, Germany; somaida@staff.uni-marburg.de (A.S.); imran.pharmacy@pu.edu.pk (I.T.); gambreen@yahoo.com (G.A.); ahmed.abdelsalam@pharmazie.uni-marburg.de (A.M.A.); ayoub@staff.uni-marburg.de (A.M.A.); matthias.wojcik@pharmazie.uni-marburg.de (M.W.); 2 Punjab University College of Pharmacy, University of the Punjab, Lahore 54000, Pakistan; 3 Department of Pharmaceutics and Industrial Pharmacy, Faculty of Pharmacy, Al-Azhar University, Assiut 11865, Egypt; 4 Department of Pharmaceutics and Industrial Pharmacy, Faculty of Pharmacy, Zagazig University, Zagazig 44519, Egypt; 5 Department of Biochemistry, Faculty of Science, University of Dschang. P.O Box. 67 Dschang, Cameroon

**Keywords:** cancer, *Xylopia aethiopica*, *Imperata cylindrical*, *Echinops giganteus*, *Dorstenia psilurus*, cell proliferation, metastasis

## Abstract

In this study, the potential cytotoxicity of four plant extracts originated from Cameroon: *Xylopia aethiopica* (XA), *Imperata cylindrica* (IC), *Echinops giganteus* (EG) and *Dorstenia psilurus* (DP) were examined in vitro. We tested the anti-proliferative activity of the methanolic extracts of these compounds using MTT assay on seven different human cancer cell lines: HeLa, MDA-MB-231, A549, HepG2, U-87, SK-OV-3 and HL60. Induction of cell death was assessed by cell cycle analysis, apoptosis was determined by Annexin V-FITC binding and caspase 3/7 activity. As well, changes in mitochondrial membrane potential (MMP) and cell migration were tested. The genetic toxicity, using the alkaline comet assay, was evaluated. The studied extracts inhibited the cell proliferation of all tested cancer cell lines with concentration dependent effect over time. All of these extracts mainly induced apoptosis of HeLa cells by the accumulation of hypodiploid cells in the sub-G0/G1 phase and increasing the activity of caspase 3/7, as well they showed potential MMP disturbance and expressed a marked inhibitory effect on cell migration. Assessment of probable genetic toxicity by these extracts revealed no or minimum incidence of genetic toxicity. Therefore, the studied plant extracts are exhibiting potent anticancer activity based upon marked induction of tumor-cell death.

## 1. Introduction

Cancer is the most serious disease worldwide that is expected to increase due to the adoption of behaviors and lifestyle factors known to cause cancer [1]. Toxicity and tumor resistance are currently the main limitations in using chemotherapeutic agents in cancer treatment. So, the discovery of new and safe treatment options is considered a big challenge [2]. Natural products represent a great source for screening new and safe anti-cancer products [3]. Plant extracts are rich in bioactive molecules based on the variety of their chemical constituents such as flavonoids, polyphenols and alkaloids which play a highly significant role in drug discovery and development process [4].

As medicinal plants represent a good alternative, especially in developing countries, many of them are used in Africa for treatment of various diseases. *Xylopia aethiopica* (XA) which is a member of the family Annonaceae, is used as a spice in Western and Central Africa, as well as to treat bronchitis, headache and ulceration [5]. In addition to its anti-diabetic effect [6], anti-anaphylactic and anti-inflammatory activities [7], several studies have shown that XA extracts possess antibacterial and antifungal activities [8,9,10,11]. *Imperata cylindrica* (IC) (family Poaceae) also known as spear grass in West Africa, has diuretic, anti- inflammatory and antibacterial activities [12,13]. It also shows a potent anthelmintic activity [14] and the methanolic extract of its rhizomes was reported as a significant neuroprotective against glutamate-induced neurotoxicity in primary cultures of rat cortical cells [15]. *Echinops giganteus* (EG), (family Compositae) is traditionally used as a medicinal agent mainly in Africa and Asia. It is mainly used as heart and gastric troubles spice, reducing as well asthma attacks. In previous studies, the root methanolic extract showed significant antioxidant [16], antibacterial [17], and antifungal effects [18]. The methanolic extract of EG roots also exhibited a significant activity against M. tuberculosis [19], the methanolic extract from the underground part also reported for cytotoxic activity against prostate cancer (Mia PaCa_2_) and two leukemia cells (CCRF-CEM and CEM/ADR5000) [20]. *Dorstenia psilurus* (DP), (family Moraceae) is widely used in traditional medicine and represents a great source of active constituents including flavonoids, alkaloids and phenolic compounds [21,22,23]. It has a therapeutic effect on cardiovascular disorders, snakebites, headache and stomach disorders, moreover it exhibits a potent antimicrobial activity and as its methanolic showed antibacterial activity against a panel of Gram-negative bacteria including multidrug resistant (MDR) phenotypes [13]. Recent study reported the isolation of two isoprenylated flavones from the root extract of DP that activate AMP-activated protein kinase (AMPK), stimulate glucose uptake and lower glycemia [24].

Recently, various studies were conducted for screening the cytotoxic activity of these plant extracts against a variety of cancer types and resistance. These studies have shown a promising effect of using these extracts against some cancer cell lines [25,26,27,28,29]. Despite the useful biological activity expressed by certain plants, the study of their possible toxicity remains particularly important. As some chemicals or secondary metabolites from plants are toxins like substances that may cause harmful effects to humans.

In this study, a well-established evaluation of the anticancer potential of these four plant extracts: (i) XA, (ii) IC, (iii) EG and (iv) DP was performed to assess their activities in the inhibition of cell proliferation in seven different human cancer cell lines: HeLa (cervical), MDA-MB-231 (breast), A549 (lung), HepG2 (liver), U-87 (glioblastoma, brain), SK-OV-3 (ovarian) and HL60 (leukemia). As well, an assessment of in vitro toxicity of these plant extracts was performed in non-cancerous HEK-293 cells. HeLa cells showed a higher sensitivity in cell proliferation assay upon treatment with these extracts, so further studies of the alteration and induction of cell death were investigated in HeLa cell line, by comparing the treated cells with these extracts to the untreated cells, using different assays involving cell cycle analysis, the caspase 3/7 activity and mitochondrial membrane potential (MMP). The effect of the studied medicinal plants on the inhibition of cell progression and metastasis was assessed using the wound healing assay. Furthermore, a single cell gel electrophoresis assay was performed to exclude any probable genetic toxicity upon using of these extracts in cancer treatment.

## 2. Results and Discussion

### 2.1. Cell Proliferation Assay

**Figure 1:**
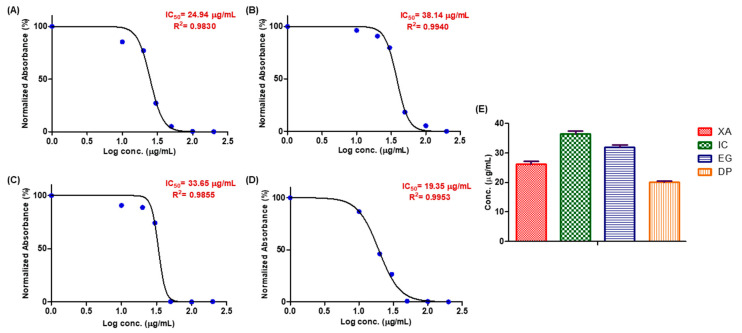
SRB assay on HeLa cells to evaluate the effect of the four plant extracts: (**A)** *Xylopia aethiopica* (XA), (**B**) *Imperata cylindrica* (IC), (**C**) Echinops giganteus (EG) and (**D**) Dorstenia psilurus (DP) on the cell growth after 48 h (X-axis: log concentrations of extracts from 1 to 200 (µg/mL) and Y-axis: the percentage of normalized absorbance). While (**E**): The comparison of average IC_50_ of the four plant extracts over 48 h (Y-axis: concentration in (µg/mL). Values are mean ± SD of three independent experiments.

The anti-proliferation effect is the first indication to be assessed when investigation novel antitumor agents, thus the cell growth inhibitory activity of the four plant extracts was initially assessed on the HeLa (cervical cancer) cell line 48 h after treatment with different concentrations of the crude methanol extracts. A dose dependent decrease in cell viability was observed (Figure 1). At a concentration of 50 µg/mL of each crude extract, DP and EG inhibited the cell growth by more than 99%, followed by XA with > 90% and IC with > 80%. The comparative IC_50_ values of XA, IC, EG and DP for HeLa cells were shown in (Figure 1E). The most potent anti-proliferative effect was induced by DP with IC_50_ value of 19.35 µg/mL, followed by XA with IC_50_ value of 24.94 µg/mL. EG and IC showed a quite similar inhibitory effect on cell growth with IC_50_ values of 33.65 and 38.14 µg/mL, respectively.

**Figure 2:**
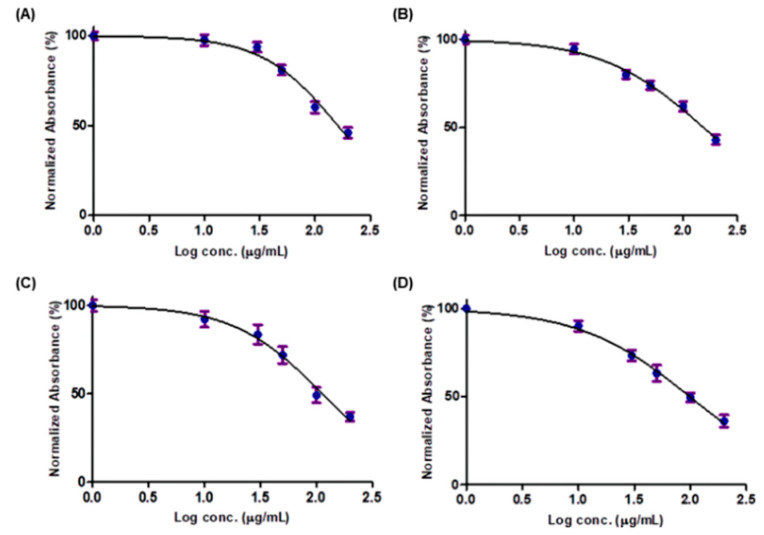
SRB assay on HEK293 cells to evaluate the effect of the four plant extracts: (**A**) XA, (**B**) IC, (**C**) EG and (**D**) DP on the cell growth after 48 h (X-axis: log concentrations of extracts from 1 to 200 (µg/mL) and Y-axis: the percentage of normalized absorbance). Values are mean ± SD of three independent experiments.

On the other hand, after 48 h treatment of the non-tumor HEK293 cells with 50 µg/mL of each crude methanol extract, there was only a negligible effect on the cell growth. The cell viability was 80.90%, 73.88%, 71.89% and 70.26% after cell treatment with XA, IC, EG and DP extracts, respectively. Only for concentrations above 100 µg/mL, a decrease by more than 50% in cell viability could be seen (Figure 2). Upon treatment of cells with a concentration of 100 µg/mL of EG and DP, cell viability decreased to 49.16% and 49.44%, respectively. While at a higher concentration of 200 µg/mL of XA and IC extracts, cell viability decreased to 48.05% and 47.30%, respectively. However a concentration of below 30 µg/mL could be considered safe for healthy cells with cell viability more than 90%.

These findings were in agreement with a recent study that was performed to assess the in vivo toxicological effect of the IC crude extract [30]. This study revealed the safety of crude methanol extract of IC at low doses on animal models, while prolonged use was suggested to be discouraged at high doses.

### 2.2. Cytotoxicity of the Plant Extracts

**Figure 3:**
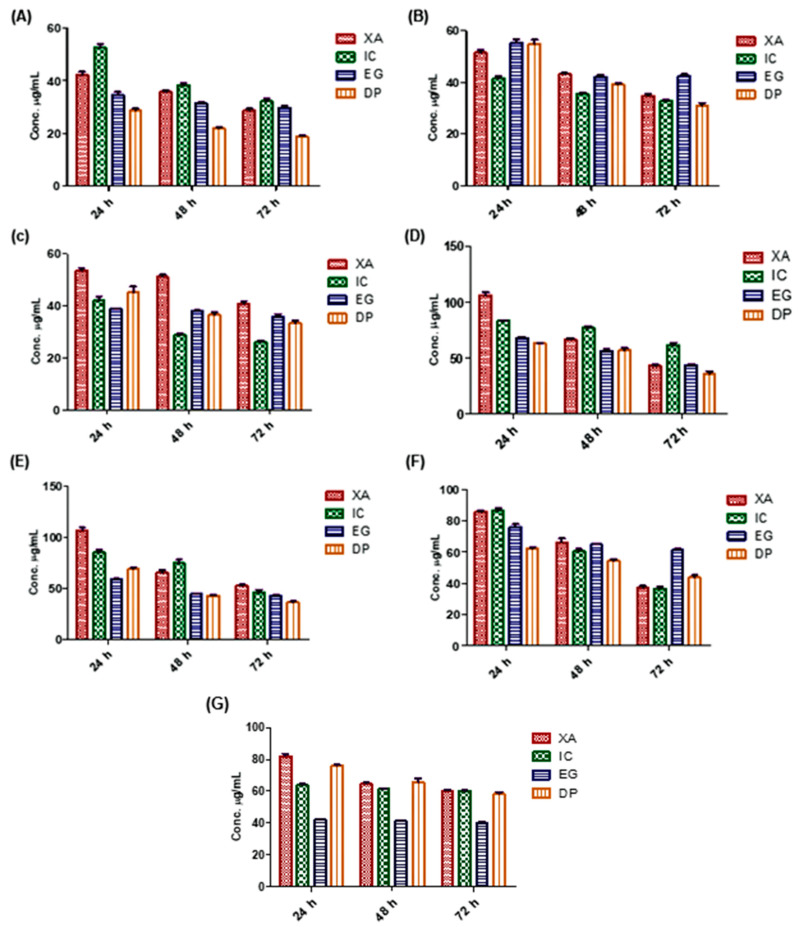
The cytotoxicity effect of the studied plant extracts on different cell lines over 24, 48 and 72 h using MTT assay: (**A**) HeLa, (**B**) HepG2, (**C**) MDA-MB-231, (**D**) A549, (**E**) SK-OV-3, (**F**) U-87 and (**G**) HL60, (Y-axis: the concentration of IC_50_ in µg/mL). Values are mean ± SD of three independent experiments.

Screening of the cytotoxicity effect of the four studied plant extracts was performed using MTT viability assay. Seven cancer cell lines were incubated with the four extracts for 24, 48 and 72 h and the status of cell growth was observed (Figure 3). The results showed that all of the aforementioned extracts expressed concentration dependent cytotoxic effects against the treated cell lines. Results confirmed that DP showed the most potent antitumor activity against cervical cancer (HeLa) cancer after 72 h, among the four studied extracts, with average half-maximal inhibitory concentration (IC_50_) values of 17.93, 21.61 and 28.27 µg/mL over 72, 48 and 24 h, respectively and 37.19 µg/mL. While IC expressed a higher cytotoxic activity against breast cancer (MDA-MB-231) after 72 h with IC_50_ of 27.30 µg/mL. EG expressed the most potent activity in growth inhibition of leukemia (HL60), while XA against glioblastoma brain cancer (U-87) with IC_50_ of 41.05 and 35.45 µg/mL, respectively. In lung cancer cells (A549), DP and XA were shown the most potent effect of growth inhibition after 72 h with IC_50_ of 39.96 and 40.79 µg/mL respectively. While the inhibitory effect of the studied plant extracts after 48 h against ovarian cancer (Sk-OV-3) cells was shown that DP and EG were more potent among the four extracts with IC_50_ value of 43.86, 44.38 µg/mL, respectively. The four extracts have possessed closely antitumor activity against hepatocellular carcinoma (HepG2) with a range from 32.99 to 53.21 µg/mL over 72 h. 

In comparison with previous studies on a variety of Cameroonian plant extracts [25,26,27,28,29], these findings confirm the potential anticancer activity of these four extracts in these seven cancer cell lines with concentration dependent effect over time. However, the most potent cytotoxic activity was against HeLa, HepG2 and MDA-MB-231 cells.

According to the US National Cancer Institute (NCI) plant screening program, a crude plant extract is generally considered to have acceptable in vitro cytotoxic activity if the IC_50_ value is less than 20 µg/mL after incubation 48 and 72 h treatment of cancer cell lines [31]. Based on these results, the four plant extracts were shown potent cytotoxic activity against HeLa cells after 72 h, with IC_50_ values of 17.93, 27.61, 30.10 and 32.29 µg/mL for DP, XA, EG and IC, respectively. Thus, HeLa cells were selected for the assessment of inhibition mechanisms.

### 2.3. Live/Dead Staining

**Figure 4:**
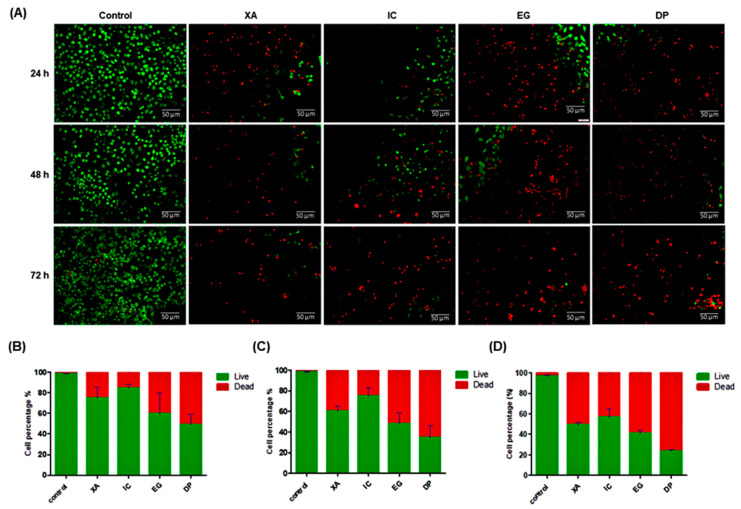
A live/dead assay after treatment of HeLa cells with the four studied extracts over 24, 48, and 72 h. (**A**) Green fluorescence denotes viable cells stained with calcein-AM, while red fluorescence represents dead cells stained with propidium iodide. Then, data analysis was performed using ImageJ software and Prism 5 (**B**) 24 h, (**C**) 48 h and (**D**) 72 h. All results are expressed as a total percentage of viable and dead cells from four random fields with mean ± standard deviation (SD) of three independent determinations. Scale bar represents 50 μm.

A live/dead assay was performed to confirm the cytotoxic activity of the four plant extracts against HeLa cell line. Cells were treated with a concentration of 30 µg/mL of each crude extract over 24, 48 and 72 h. Then, dual staining with fluorescence dyes was performed using calcein-AM and propidium iodide. The results showed a significant decrease in viable cells after being with the extracts (Figure 4A). Data confirmed the cytotoxic activity of these extracts with time dependent manners. As the percentage of viable cells of untreated cells was 97.10 ± 0.8% after 72 h, while the percentage of viable cells decreased to 75.36 ± 5.5%, 60.36 ± 4.3% and 49.46 ± 3.8% after cell treatment with XA over 24, 48 and 72 h, respectively. After treatment of cells with IC, viable cells decreased to 85 ± 2.4%, 75.25 ± 5.7% and 56.79 ± 4.9% over 24, 48 and 72 h, respectively. The percentage of viable cells dramatically decreased, upon treatment of cells with EG to 59.77 ± 5.9%, 48.28 ± 3.1% and 41.35 ± 2.4% over 24, 48 and 72 h, respectively. The results confirmed that DP was shown a potent cytotoxic activity towards HeLa cells, as the percentage of viable cells significantly decreased to 49.30 ± 4.1%, 34.72 ± 3.5% and 23.64 ± 1.2% over 24, 48 and 72 h, respectively (Figure 4B–D). These results suggest that the four studied plant extracts are potential cytotoxic agents for the studied cell lines. 

### 2.4. Cell Cycle Analysis

**Figure 5:**
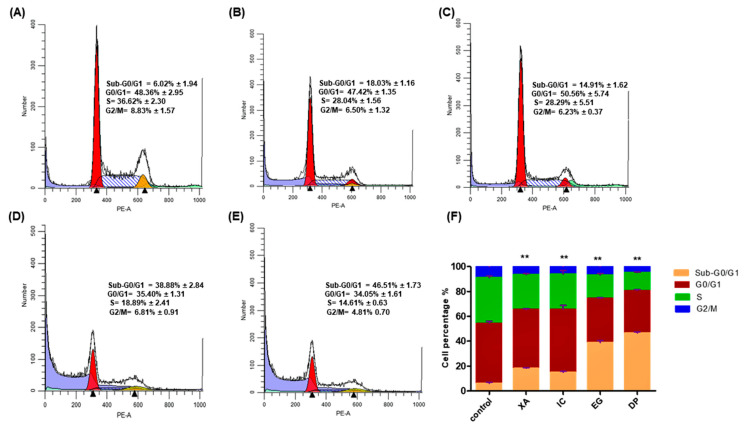
Cell cycle analysis by flow cytometry for HeLa cells treated with IC_50_ of the different studied plant extracts for 48 h: (**A**) Control, (**B**) XA, (**C**) IC, (**D**) EG and (**E**) DP. While, (**F**) Comparative analysis among different plant extracts for Sub-G0/G1, G0/G1, S and G2/M Phases. The cell cycle distribution was analyzed by ModFit LT software. All results are expressed in the histogram as total percentages of cells from four different groups with mean ± SD of three independent determinations. All data collected from experiments were performed in three replicates and analyzed using the one-way analysis of variance (ANOVA) at a significance level of *p* < 0.05 and indicated by **.

To investigate the mechanism of growth inhibition, the effect of the four studied crude extract on the cell cycle distribution of HeLa cells were analyzed after 48 h of the treatment of cells with the concentration of IC_50_ obtained from the MTT assay, followed by fixation and PI staining. The cell cycle arrest was analyzed using flow cytometry. The results in Figure 5 show an obvious alteration of the distribution of different phases. The cell population was seen to be increasingly accumulated at the SubG0/G1 phase upon treatment with the studied extracts. Compared to the untreated cells that showed a percentage of 6.02 ± 1.94% in this phase, XA and IC showed a considerable increase with percentages of 18.03 ± 1.94% and 14.91 ± 1.16%, respectively, while EG and DP significantly increased the accumulation of hypodiploid cells in the sub-G0/G1 phase with percentages of 38.88 ± 2.84% and 44.51 ± 1.73%, respectively. The data showed that there was no significant change in cell arrest in all other phases. The results suggest that HeLa cells underwent apoptosis upon treatment with the studied plant extracts.

Plant extracts exert their cytotoxic effects through common mechanisms including cell cycle arrest and cell death by apoptosis. The potential of anticancer agents is evaluated by the ability to initiate cell cycle arrest in cancer cells [32]. Production of apoptotic cells, which are resulting from DNA fragmentation, display a broad hypo-diploid sub-G0/G1 peak which is easily detected with sufficient loss of cellular DNA and can be discriminated by flow cytometry [33]. Corresponding to these findings, in the present study, the four studied extracts have the potential to cause sufficient DNA loss to induce apoptosis, as the accumulation of hypodiploid cells in the sub-G0/G1 phase is an indication of apoptotic cell death. Several intracellular cascades, such as the activation of caspases and the disruption of MMP would be a confirmation for apoptosis. 

### 2.5. Annexin V-FITC/PI for Apoptosis Detection

**Figure 6:**
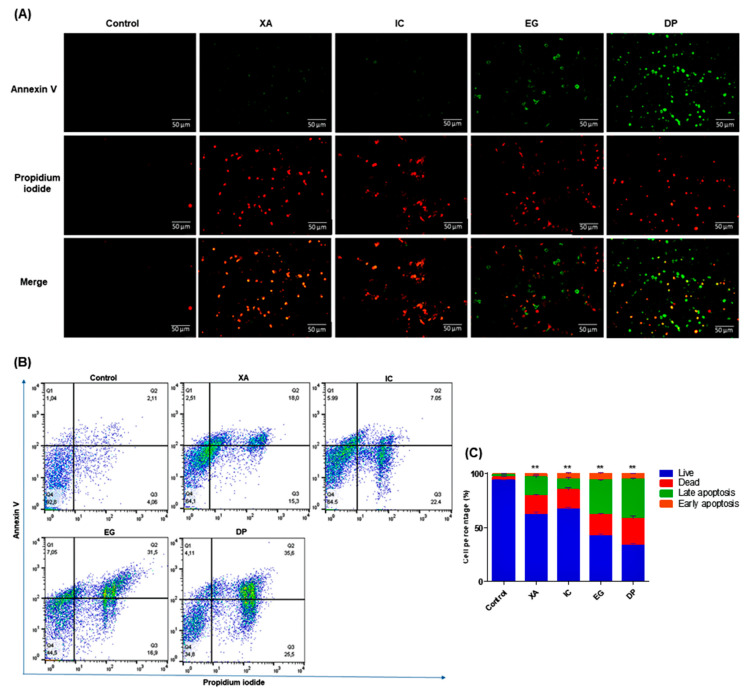
Detection of apoptosis and necrosis using Annexin V-FITC and PI dual staining after 48 h of treatment of HeLa cells with IC_50_ of the studied crude extracts XA, IC, EG and DP in comparison to untreated cells. (**A**) Images by fluorescent microscopy represent one of the three independent experiments, (**B**) Results, after flow cytometry analysis, are expressed in the histogram as total percentages of cells from four different quadrants (Q1 = early apoptosis, Q2 = late apoptosis, Q3 = necrosis, Q4 = live), with mean ± SD of three independent determinations. All results are expressed in the histogram as total percentages of cells from four different groups with mean ± SD of three independent determinations. (**C**) Bar graph for values of cell percentages, expressed in mean ± standard deviation (SD) and analyzed using the one-way analysis of variance (ANOVA) at a significance level of *p* < 0.05 and indicated by **.

In order to verify that the effect of the studied extracts on the growth inhibition of HeLa cells was related to apoptosis, analysis of the apoptotic and necrotic cells was performed using Annexin V. After 48 h of the treatment with the IC_50_ of the four extracts, a double labelling was done with PI that staining the necrotic cells with red fluorescence and Annexin V-FITC which produce cytoplasmic green labelling for apoptotic cells. Images from fluorescent microscopy (Figure 6A), showed that viable cells were negative for both PI and Annexin V. While XA and IC were shown considerable green and red labelling for both apoptotic and necrotic cells, respectively. A significant detection of apoptotic cells was shown upon cell treatment with both EG and DP, indicating that cells mainly underwent apoptosis with those extracts. 

The visual observations were confirmed by the quantitative analysis by flow cytometry. Data in Figure 6B–C shows the distribution of HeLa cells within four different quadrants (Q1 = early apoptosis, Q2 = late apoptosis, Q3 = necrosis, Q4 = live) and represent one of three sets of independent experiments conducted. Cells that are undergoing apoptosis will shift from the viable quadrant (Q4) to the early apoptosis quadrant (Q1), and eventually end up in late apoptosis quadrant (Q2). On the other hand, cells that undergo necrosis will shift from viable quadrant (Q4) to the late necrosis quadrant (Q3). Un-treated cells showed a percentage of 92.8 ± 1.06% for viable cells, 4.06 ± 0.73% for dead cells, 2.11 ± 0.30% for late apoptosis and 1.04 ± 0.09% for early apoptosis. XA and IC expressed increase in the late apoptotic population to 18 ± 2.08% and 7.05 ± 2.6%, respectively. While EG and DP showed significant increase in the late apoptosis to 31.50 ± 1.26% and 35.6 ± 1.42%, respectively. 

As well, a considerable increase was shown in necrotic cells, with percentages of 15.30 ± 2.35%, 22.40 ± 3.82%, 16.90 ± 3.36% and 25.50 ± 4.54%, upon treatment with XA, IC, EG and DP, respectively. Lastly, early apoptotic cells, represented by Q1, displayed only a slight increase in cell distribution as a result of treatment with the studied extracts XA, IC, EG and DP to percentages of 2.51 ± 0.34%, 5.99 ± 1.36%, 7.05 ± 1.23%, 4.11 ± 0.89%, respectively. Phosphatidyl-serine (PS) on the outer layer of the plasma membrane acts as a recognition site of phagocytes during the early stage of apoptosis [34]. Annexin V, a calcium-dependent protein can bind to the exposed phosphatidyl-serine (PS) on the external layer of the membrane [35]. In this study, it was observed that the percentage of cells undergoing late apoptosis increased significantly, thus confirming that apoptosis was one of the major modes of cell death induced by the four studied plant extracts, especially EG and DP extracts. 

### 2.6. Effect on the Activity of Caspase 3/7

**Figure 7:**
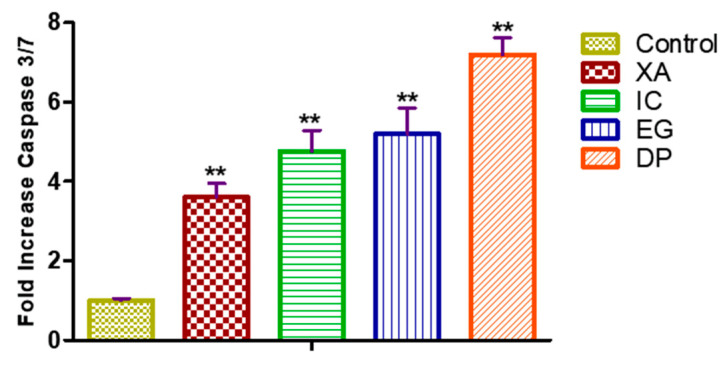
Enzymatic activity of caspase 3/7 on HeLla cells and after fold increase in activity after 24 h treatment of with IC_50_ of the four studied plant extracts XA, IC, EG and DP. All data expressed as mean ± standard deviation (SD) at a significance level of *p* < 0.05 and indicated by **.

To investigate whether the apoptosis effect induced by the four studied plant extracts is dependent upon the caspases activation, the caspase 3/7 activity examined on HeLa cells treated with the concentration of IC_50_ values of the four studied extracts for 24 h (Figure 7). DP expressed a significant effect on activation of caspase 3/7 up to 7.17 ± 0.72 fold compared to the untreated cells. As well, EG efficiently enhanced the caspase 3/7 activity by 5.15 ± 1.14 fold, while cells treated with XA and IC expressed an increase in the activities of caspase 3/7 by 3.60 ± 0.58 and 4.75 ± 0.92 fold compared to the untreated cells that expressed only 1.12 ± 0.19 fold increase in activity. 

One of the important measures that play essential roles in apoptosis, necrosis, and inflammation is caspases which are a family of cysteine proteases, causing cleavage of cellular protein [36]. Examination of the activities of caspase 3/7 in HeLa cells with the four studied plant extracts has shown a marked increase in the activity of caspase 3/7, which confirm the effect of these extracts in apoptotic cell death as observed previously in cell cycle analysis and Annexin V.

### 2.7. Effect on Mitochondrial Membrane Potential (MMP)

**Figure 8:**
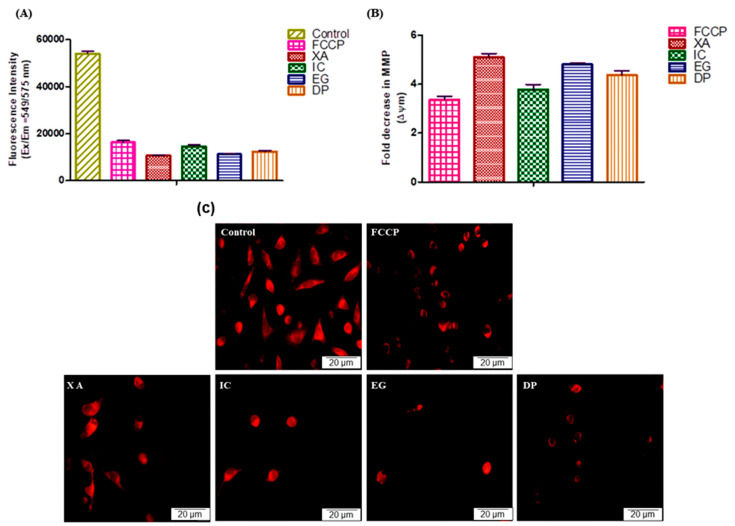
Effect of the IC_50_ values of the four plant extracts and 50 µM positive control (FCCP) on the MMP of HeLa cells after 24h. Un-treated cells representing the negative control. (**A**) Fluorescence Intensity after 24 h treatment, (**B**) Analysis of fold Change of MMP. Values expressed in mean ± standard deviation (SD). (**C**) Fluorescence Images after 24 h treatment, visualize the breakdown of MPP after treatment of HeLa cells with the IC_50_ of the four studied plant extracts, followed by staining by 500 nM of TMRE.

As the disruption of the MMP is one of the sequential events exhibited during the apoptotic pathway, the effect of the four studied plant extracts in this alteration after 24 h of treatment against HeLa cells was compared to the positive control FCCP (Figure 8A) with untreated cells representing the negative control. After the cells were treated with IC_50_ of XA and EG, the MMP closely dropped to 19.70% and 20.84%, respectively. As well, DP and IC disrupted the MMP to 23.02% and 26.90%, while FCCP, the positive control for the MMP breakdown, altered the MMP to 30.20% compared to the control cells which represent the 100%. The results were analyzed in Figure 8B to assess the fold change in MMP after the treatment of different studied plant extracts. The data showed that all plant extracts expressed a significant alteration of the MMP. XA revealed a significant alteration decrease by 5.09 ± 0.21-fold, followed by EG, DP and IC with 4.80 ± 0.12, 4.37 ± 0.43 and 3.77 ± 0.63-fold, respectively. Comparatively, FCCP altered the MMP with a 3.35-fold decrease. Further analysis using fluorescence microscopy (Figure 8C) was performed to visualize the breakdown of MPP after treatment of HeLa cells with the IC_50_ of the four studied plant extracts, followed by staining with 500 nM of tetramethylrhodamine ethyl ester (TMRE.) The results represent the significant depletion of MMP after cells were treated with the four plant extracts, compared to the positive control (FCCP) and the untreated cells, as the remaining living cells obviously showed a depolarization of MMP which induced the apoptotic pathway and cell death. 

Mitochondria play an essential role in the physiological metabolism of cells and energy supply for cell survival [37]. Mitochondrial membrane potential (MMP) is one of the sequence processes that integrated during apoptotic pathway. Results in this study revealed that the four studied plant extracts showed significant disruption of the MMP compared to the untreated. These findings confirm that cells undergo apoptosis after treatment with these extracts by potential MMP alteration which represent is a key step in the intrinsic apoptotic pathway

### 2.8. Wound Healing Assay

**Figure 9:**
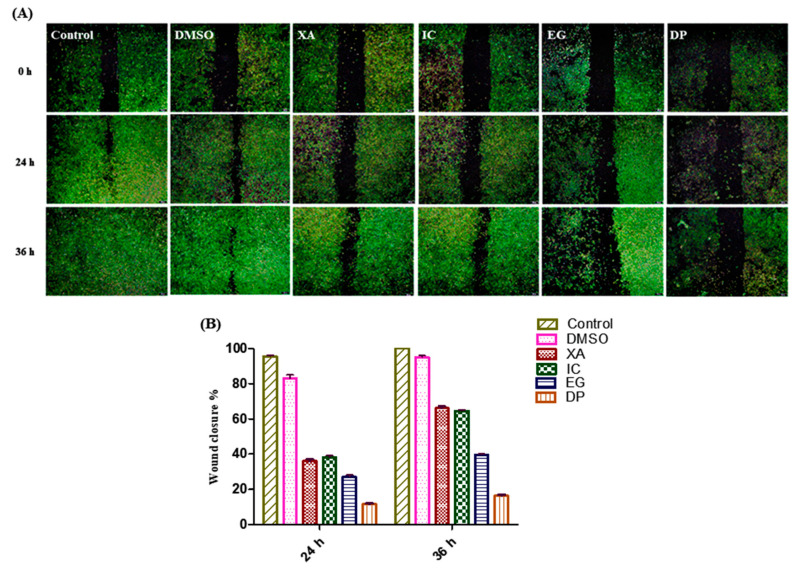
Effect of the plant extracts on cell growth; Scratching was performed using a 200 µL pipette tip. (**A**) Metastatic progression by scratch test. While, (**B**) Time dependent cell wound closure % under the influence of the four plant extracts XA, IC, EG and DP compared to control (un-treated cells) and cells treated with vehicle (0.1% DMSO) as negative control. Values expressed in mean ± standard deviation (SD).

Assessment of the inhibitory effect of the four studied plant extracts on the progression and migration was performed against HeLa cells over 24 and 48 h. the negative control is represented by un-treated cells and cells treated with vehicle (0.1% DMSO). The treatment of cells with the IC_50_ of the studied plant extracts resulted in a significant blocking the progression and wound healing of the scratch area compared to the untreated cells and control cells treated with the solvent (0.1% DMSO) as a control (Figure 9A). Data analysis of the percentage of the wound closure was performed and data showed that the percentage decreased at 24 h from 96% and 81% for untreated cells and DMSO, respectively to 38%, 36%, 27% and 11.5% for IC, XA, EG and DP. After 36 h, the scratch area for the untreated cells was totally covered by cells with a percentage of 100% and for cells treated with DMSO by 95%. However, the significant effect on the scratch area, which continued up to 36 h, was observed in cells treated with DP where the percentage of wound closure was only 16.5% followed by 39.5% for cells treated with EG. While the coverage area of the scratch increased to 64.5% and 66% for cells treated with IC and XA, respectively (Figure 9B), from these findings it was obvious that the four studied plant extracts exhibited a potential inhibitory effect of metastatic progression. 

Cell migration in vitro, or cell metastasis in vivo, represents one of the main features of malignant tumors that leads to increase in the mortality rate in cancer disease [38]. In this study, wound-healing assay showed that the four studied plant extracts significantly inhibited the cell migration in HeLa cells when treated with the IC_50_ values. DP and EG showed more potential and continuous effect in the inhibition of wound closure up to 36 h. These findings could represent potent anticancer agents that inhibited cancer metastasis.

### 2.9. Single Cell Gel Electrophoresis Assay

To assess if there is any possible toxicity induced by the studied plant extracts especially damage to the DNA and genotoxicity, the comet assay was performed. The principle of the comet assay is based on the fact that DNA strands which occur as a negatively charged supercoiled structure in the nucleus can be fragmented due to the exposure to the toxins or drug treatments [39].

**Figure 10:**
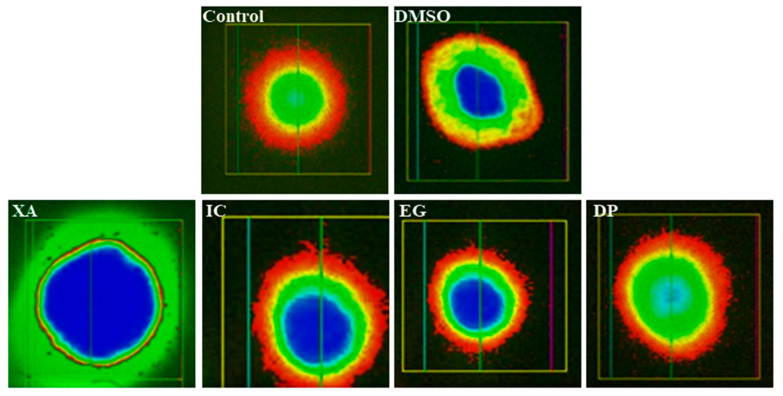
Comet assay: fluorescence micrographs of genotoxicity to Hela cells obtained after 24 h of the treatment of the four studied plant extracts XA, IC, EG and DP compared to untreated cells and vehicle (0.1 DMSO) treated cells.

According to Figure 10, it can be observed that no direct DNA strand breakage was caused by IC_50_ levels of the three of the studied plant extracts—IC, EG and DP—compared to the untreated cells and vehicle control (0.1% DMSO). While IC_50_ of the plant extract XA might interfere with low DNA damage. Several parameters including percentage of DNA in comet tail (% tail-DNA), tail length (TL), and tail moment (TM) were used in the past to monitor DNA strand-breakage with the comet assay. In this study, the percentage of tail-DNA was used to quantify DNA strand-breakage in Hela cells after 24 h of treatment with the plant extracts. Data in Table 1, confirmed the results as IC, EG and DP exhibited percentage (% tail-DNA) <10% which represent the value for undamaged nuclei, while XA shown value (% tail-DNA) of 19.04 ± 2.10% that indicate low-damaged nuclei [40]. From these results, it can be assumed that these plant extracts can be used in in-vivo studies with no or minimum incidence of genetic toxicity.

## 3. Materials and Methods

### 3.1. Materials

3-(4,5-Dimethylthiazol-2yl)-2,5-diphenyltetrazolium bromide (MTT), tetramethylrhodamine ethyl ester (TMRE), sulphorhodamine B (SRB), trichloroacetic acid (TCA), Tris base (tris [hydroxymethyl]aminomethane), calcin AM, annexin V and propidium iodide (PI) were obtained from Sigma Aldrich Chemie GmbH (Taufkirchen, Germany). Dimethyl sulfoxide (DMSO) was procured from Carl Roth GmbH & Co. (Karlsruhe, Germany). Roswell Park Memorial Institute (RPMI) 1640 Medium, Dulbecco’s modified Eagle’s minimum essential medium (DMEM), Fetal bovine serum (FBS) were purchased from Capricon Scientific (Ebsdorfergrund, Germany). The Caspase-Glo 3/7 Assay kit was procured from Promega (Walldorf, Germany), and carbonyl cyaninde 4-(trifluoromethoxy) phenylhydrazone (FCCP) was from Cayman Chemical (Ann Arbor, Michigan, USA).

### 3.2. Plant Material and Extraction

**Figure 11:**
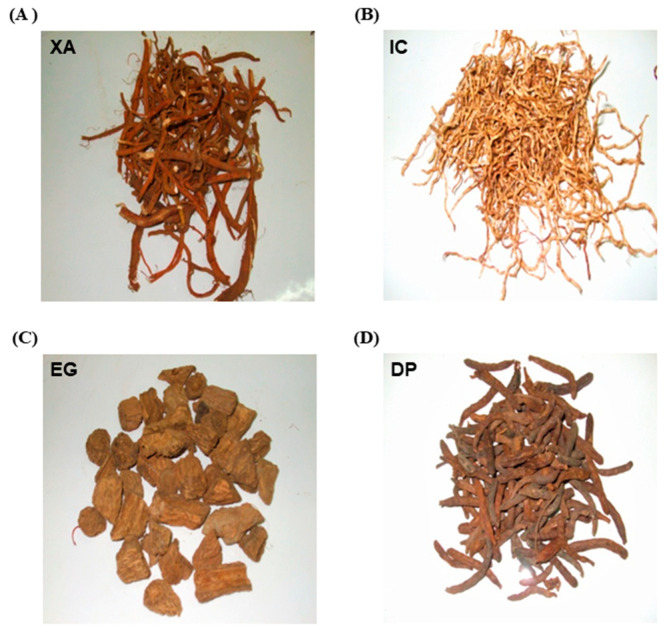
Morphological characteristics of the studied plants: (**A**) XA, (**B**) IC, (**C**) EG and (**D**) DP.

Extraction of the four studied plants: *Xylopia aethiopica* (XA), *Imperata cylindrica* (IC), *Echinops giganteus* (EG) and *Dorstenia psilurus* (DP) (collected in Cameroon) were performed at our partner laboratory at the University of Dschang, Cameroon. Plant materials were purchased from Dschang local market, West Region of Cameroon in August 2018. They were identified at the Cameroonian National Herbarium where voucher specimens were deposited under the flowing reference number (Table 2, Figure 11). The extraction was done by maceration of 100 g of the plant material in 500 mL methanol for 48 h, then, the methanolic extracts were concentrated by rotary evaporation under reduced pressure to obtain the crude extracts. The extracts were then conserved at 4 °C until further use.

### 3.3. Cell Lines and Cell Culture

The HEK293 cell line (human embryonic kidney) and the human cancer cell lines: HeLa (cervical), MDA-MB-231 (breast), A549 (lung), HepG2 (liver), U-87 (glioblastoma, brain), SK-OV-3 (ovarian) and HL60 (acute promyelocytic leukemia) were obtained from the American Type Culture Collection (ATCC, Manassas, VA, USA). The cells were grown in recommended culture media supplemented with 10% FBS and MEM-non-essential amino- acids (Gibco^TM^ Thermo-Fisher, Waltham, MA, USA), 50 µg/mL gentamicin and 2.5 µg/mL amphotericin B in a 5–7% CO_2_ humidified atmosphere at 37 °C. The cells were grown as a monolayer for no more than 15 passages.

### 3.4. SRB Assay

To initially assess the possible cytotoxic effect of the studied plant extracts, the colorimetric sulphorhodamine-B (SRB) assay was used for measurement of cell proliferation [41]. Briefly, 4 × 10^4^ of HeLa (cervical cancer) and HEK293 (non-tumor) cells were added to each well of a 96-well plate and incubated overnight to allow for cell attachment. The cells were then treated with serial dilutions of the four plant extracts: XA, IC, EG and DP (200 to 1 µg/mL) and 1% Triton-x was used as a positive control. Untreated cells receiving the same volume of medium were served as a control while the concentration of vehicle control (DMSO) was kept at or below 0.1%. After 48 h exposure, the cells were fixed with ice-cold 10% TCA at 4 °C for 1 h, then washed four times under slow-running tap water, afterwards, stained with 0.057% (*w*/*v*) SRB in 1% acetic acid, washed and air-dried. Bound dye was solubilized with 200 μL of 10 mM Tris base solution (pH 10.5). The plates were read at 540 nm absorbance on a Fluostar microplate reader (BMG Labtech, Ortenberg, Germany), the determination of 50% inhibitory concentration (IC_50_) was based on dose-response curves between the extract concentration and percent growth inhibition using the GraphPad Prism 5 software. The values are expressed as mean ± SD with all the experiments independently performed in triplicate.

### 3.5. Cell Viability Assay

Cell viability was measured by a standard MTT assay method [42]. The plant extracts were dissolved in DMSO, then diluted with cell culture medium to different serial dilutions (200 to 1 µg/mL) while the concentration of vehicle control (DMSO) was kept at or below 0.1%., 1% Triton-x was used as a positive control and untreated cells receiving the same volume of medium served as control For all adherent cells, the cells were cultured in 96-well plates (4 × 10^4^ cells per well) and incubated at 37 °C for 24, 48 and 72 h. After that, a 20 μL MTT (5 mg mL^−1^) solution was added to each well and incubated for 4 h, then 150 μL of DMSO was added to each well to dissolve the formazan crystals. The absorbance data was detected by a microplate reader at 490 nm. For Leukemia HL60, a suspension of 4 × 10^4^ cells/mL were seeded in 96-well plates and immediately after, serial dilution of the extracts in the media was added. After adequate incubation 24, 48 and 72 h, the 96-well plates were centrifuged at 1000 × g, 4 °C for 5 min in a microplate-compatible centrifuge, then the media was carefully aspirated and treated with MTT solution as above mentioned. The viability was observed based on comparison with the absorbance of untreated and treated cells. The IC_50_ values were obtained from the concentration of the plant extracts that induced 50% inhibition of cell growth using the GraphPad prism 5 software. Additionally, each test was replicated three times independently.

### 3.6. Live Dead Staining

Assessment of cell viability of HeLa cells upon treatment with the four studied extracts was conducted using the LIVE/DEAD^®^ Viability/Cytotoxicity Kit. A total of 2.0 × 10^5^ cells/well were plated on the surface of a sterile glass coverslip placed in a twelve-well plate and incubated overnight before treatment with a concentration of 30 µg/mL of the crude extracts. Cells were washed with 1× PBS solution before staining using a dual-fluorescence of calcein-AM (2.0 µM) and propidium iodide (20 µg/mL). Cell washed twice after 15 min with 1 × PBS solution, then visualization of samples was carried out using a fluorescence microscope (CKX-53 Olympus, Tokyo, Japan). Viable cells stained with green fluorescence and dead cells labelled with red fluorescence correlating to calcein-AM and Propidium iodide, respectively. Four random fields of view for each sample were captured and were analyzed using ImageJ software for three independent experiments. The percentages of viable cells were calculated as follows:

Viable cells (%) = [live cells/(live cells + dead cells)] × 100(1)

### 3.7. Cell Cycle Analysis

A total of 3 × 10^5^ cells were seeded into a well of a 12-well plate and incubated with the IC_50_ values obtained from the MTT assay of the four studied plant extracts and untreated cells as a control for 24 h. Following incubation, cells were trypsinized, washed with PBS and resuspended in 100 µL of ice-cold PBS. Cells transferred to a 2 mL sample tube and shaking at 800 rpm while adding 900 µL of ice-cold 70% ethanol drop-wise then incubated at −20 °C for 2 h. Cells were further centrifuged for 5 min at 8000 rpm, 4 °C, cell pellets were resuspended in 1 mL ice-cold PBS and the centrifugation was repeated then pellets were re-suspended in 1 mL of staining solution (PBS, 0.01% tween 80, 20 µg/mL PI, 1 µL/mL RNase [100 mg/mL]) and incubated at 37 °C for 30 min [43]. After washing and centrifugation, the cell suspension was vortexed and filtered using a polystyrene round bottom tube equipped with a cell strainer cap. The cell cycle distribution was then analysed using PE-A channel with a flow cytometer (BD Biosciences LSR II FACS, San Francisco, CA, USA) and 10,000 events per sample were acquired. The percentages of cells in the different cell cycle phases were analyzed in triplicate by the ModFit LT software (version 5.0).

### 3.8. Annexin V-FITC/PI for Apoptosis Detection

Further analysis for apoptosis was performed using a FITC Annexin V Apoptosis Detection Kit according to the manufacturer’s instructions. Briefly, a total of 5.0 × 10^5^ of HeLa cells were cultured before being treated with the IC_50_ values, obtained from the MTT assay of the studied crude extracts, for 48 h. For the qualitative analysis, cells were seeded on the surface of a sterile glass coverslip placed in a twelve-well plate and incubated overnight before treatment. Treatment-free cells were grown as negative controls. Then, cells were washed twice with 1× binding buffer and further incubated for 15 min with 200 µL of the binding buffer containing Annexin V-FITC and PI in the dark. Visualization of samples was carried out using a fluorescence microscope (CKX-53 Olympus, Tokyo, Japan). Apoptotic cells stained with green fluorescence and dead cells labelled with red fluorescence correlating to annexin V-FITC and propidium iodide, respectively. Four random fields of view for each sample were captured.

For the quantitative analysis, the cells were harvested with trypsin and centrifuged at 300 × g for 10 min after treatment. Cells were then washed twice with 1 × PBS buffer and further incubated with 100 µL of the binding buffer containing annexin V-FITC and PI in the dark for 15 min. After that the samples were mixed with 400.0 µL binding buffer before being analyzed using a flow cytometer (BD Biosciences LSR II FACS). Results for three independent experiments expressed in a scatter plot as four different quadrants representing viable cells, necrosis, early and late apoptosis.

### 3.9. Caspase-Glo 3/7 Activity

The influence of extracts on caspase 3/7 activity in HeLa cells was observed using Caspase-Glo 3/7 Assay kit (Promega). Following the manufacturer protocol, cells cultured in RPMI were seeded in 96-well plates overnight, then treated with the IC_50_ of the crude plant extracts obtained from the MTT assay and untreated cells as a control. After 24 h treatment, 100 µL of caspase reagent were added to each well, mixed and incubated for 1 h at room temperature. The luminescence was measured using a Fluostar Optima microplate reader (BMG Labtech, Ortenberg, Germany), then caspase activity was expressed as percentage of the untreated control within five replicates reading.

### 3.10. Analysis of Mitochondrial Membrane Potential MMP (ΔΨm)

The mitochondrial membrane potential assay was performed by using tetramethylrhodamine ethyl ester (TMRE) to label active mitochondria. Simply, HeLa cells were seeded in 96-well plates (4 × 10^4^) cells per well), after incubation overnight, cells were treated with IC_50_ of the four plant extracts obtained from the MTT assay and untreated cells worked as negative control. 24 h later, 50 µM of carbonyl cyanide 4-(trifluoromethoxy) phenylhydrazone (FCCP) was added to one sample replicate as a positive control for 20 min, then cells were treated with 500 nM of TMRE and incubated for 20 min at 37 °C. Cells were washed twice with 0.2% bovine serum albumin (BSA) *w*/*v* in PBS, then fluorescence was detected using a Flurostar Optima microplate reader (λ_Ex_/λ_Em_ = 549/575 nm). For further evaluation of the disruption of MMP, visualization of the MMP analysis was done under a fluorescence microscope (CKX-53 Olympus). All experiments were performed in independent triplicates.

### 3.11. Wound Healing Assay

Inhibition of cell migration and metastasis was evaluated by wound healing assay [44]. HeLa cells were seeded in 24 well plates. After 24 h, cells were treated with concentration values of IC_50_ of the different plant extracts obtained from the MTT assay and 0.1% DMSO (solvent control) for 2 h in serum-free medium. A scratch was made with a 200 µl pipette tip. Cells were then washed twice with ice-cold PBS (pH 7.4) and fresh medium was added. Wound closure was observed immediately (0 h) using an inverted microscope (CKX53, Olympus) and at different time intervals (24 h and 36 h). Cell migration and percentage wound healing were also calculated using Sketch and CalcTM^®^ along with Gimp2.10.10^®^ software measuring the distance between wound closures. The experiment was independently performed in triplicates.

### 3.12. Single Cell Gel Electrophoresis

The assessment of the DNA damage and genotoxicity induced by the studied plant extracts was performed by the single-cell gel electrophoresis (Alkaline Comet Assay). All the procedures were performed in dark [45]. Briefly, 1 × 10^5^ HeLa cells per well were seeded into a six-well plate and were allowed to adhere overnight. Then cells were treated with IC_50_ of the different plant extracts obtained from the MTT assay and 0.1% DMSO (solvent control) for 24 h. Then the cells were trypsinized and centrifuged for 5 min at 1000 rpm to get the cell pellet. The obtained cell suspension was washed twice using sterile PBS (pH 7.4) and cell density was adjusted accordingly. After that, 80,000 cells (25 µL) of the treated and untreated cell suspension was mixed with 75 µL of 1% of pre-warm low melting agarose (LMA) (Carl Roth GmbH). The mixture was applied on the super frost glass slide previously pre-coated with 1% standard normal melting agarose (NMA) and was immediately covered with coverslips. The glass slides were then placed on an ice block for 10 min until solidified and the coverslips were gently removed. The cell membrane lysis was done by submerging the slides overnight into the staining jar containing cold lysis solution (300 mM NaOH, 1.2 M NaCl, 2% DMSO and 1% Triton X-100) [39]. The slides were then transferred to the electrophoresis tank containing alkaline electrophoresis buffer (300 mM NaOH and 1 mM EDTA) and were left in the buffer for 30 min to allow the unwinding of DNA. Electrophoresis was performed for 30 min at 250 mA current and 25 V, resulting in the DNA unwinding and exposing the alkali labile sites. After the electrophoresis, the slides were neutralized by washing the slides with double distilled water. The cell fixation was then done by submerging the slides into the 70% ethanol for 20 min. After fixation, cells were stained with SYBR^®^ safe DNA staining dye (1:10,000 in PBS) for 20 min. Finally, the slides were washed with double distilled water to remove any unbound stains. The comet analysis was done under a fluorescence microscope (CKX-53 Olympus). Fifty individual comets were scored for each sample and analyzed using Comet Assay IV^®^ software.

### 3.13. Statistical Analysis

Non-linear curve fitting functions were applied on normalized dose-response cell viability data obtained from SRB and MTT assays, then IC_50_ values were calculated using GraphPad Prism 5. All the experiments were performed in triplicate unless otherwise stated and results are expressed as mean ± SD. One-way ANOVA was performed on data obtained of bar graphs using GraphPad Prism 5. Significance levels of *p* < 0.05 were considered for the rejection of the null hypothesis. 

## 4. Conclusions

In this study, the cytotoxic activity of extracts from four Cameroonian plants—XA, IC, EG and DP—was examined. These extracts could represent potential antitumor agents against the examined cancer cells in a concentration dependent manner. Cell cycle analysis showed the accumulation of hypodiploid cells in the sub-G0/G1 phase which is considered to be a marker for apoptotic cell death. As well, apoptosis was induced, by all of these extracts, with an increase in the caspase 3/7 activity and significant MMP disruption. These extracts introduce a promising option for inhibition of cancer cell metastasis. Further studies are warranted to identify the active constituents which are responsible for the anticancer properties and assessment of the dose-response relationship in vitro and in vivo.

## Figures and Tables

**Table 1:** Percentage of the head and tail of DNA after cell treatment with IC_50_ of studied extracts.

	Head DNA (%) ± SD	Tail DNA (%)
Control	99.78 ± 0.07	0.21
DMSO	94.63 ± 1.09	5.36
*Xylopia aethiopica* (XA)	80.96 ± 2.10	19.04
*Imperata cylindrica* (IC)	91.82 ± 1.34	8.17
*Echinops giganteus* (EG)	96.49 ± 0.79	3.5
*Dorstenia psilurus* (DP)	93.11 ± 1.33	6.89

**Table 2:** The botanical origin of the four studied plant extracts.

Scientific Name	Family	Parts Used	Voucher Number
*Xylopia aethiopica (Dunal) A. Rich*	Annonaceae	fruits	16419/SRF-Cam
*Imperata cylindrica* (L.) Raeusch.	Poaceae	roots	30139/SRFK
*Echinops giganteus* A. Rich.	Compositae	roots	23647/SRF-Cam
*Dorstenia psilurus* Welwitch	Moraceae	roots	44839/HNC
